# In Vitro Utilization of Prebiotics by *Listeria monocytogenes*

**DOI:** 10.3390/microorganisms12091876

**Published:** 2024-09-11

**Authors:** Tereza Kodešová, Anna Mašlejová, Eva Vlková, Šárka Musilová, Kristýna Horváthová, Hana Šubrtová Salmonová

**Affiliations:** Department of Microbiology, Nutrition and Dietetics, Faculty of Agrobiology, Food and Natural Resources, Czech University of Life Sciences Prague, Kamýcká 129, 165 21 Prague, Czech Republic; kodesovat@af.czu.cz (T.K.); liskovaanna@af.czu.cz (A.M.); vlkova@af.czu.cz (E.V.); musilovas@af.czu.cz (Š.M.); horvathovak@af.czu.cz (K.H.)

**Keywords:** *Listeria monocytogenes*, prebiotics, oligosaccharides, beta-(1,3)-D-glucan, pathogen growth

## Abstract

*Listeria monocytognes* is an emerging pathogen responsible for the serious foodborne disease, listeriosis. The commensal gut microbiota is the first line of defense against pathogen internalization. The gut microbiome can be modified by prebiotic substrates, which are frequently added to food products and dietary supplements. Prebiotics should selectively support the growth of beneficial microbes and thus improve host health. Nevertheless, little is known about their effect on the growth of *L. monocytogenes*. The aim of this study was to evaluate the growth ability of four *L. monocytogenes* strains, representing the most common serotypes, on prebiotic oligosaccharides (beta-(1,3)-D-glucan, inulin, fructooligosaccharides, galactooligosaccharides, lactulose, raffinose, stachyose and 2′-fucosyllactose and a mixture of human milk oligosaccharides) as a sole carbon source. The results showed that only beta-(1,3)-D-glucan was metabolized by *L. monocytogenes*. These cell culture data suggest that beta-(1,3)-D-glucan may not be selectively utilized by healthy commensal bacteria, and its role in intestinal pathogen growth warrants further exploration in vivo.

## 1. Introduction

*Listeria monocytogenes* is a ubiquitous Gram-positive, facultative anaerobic, non-spore-forming bacterium. This organism is an opportunistic intracellular pathogen responsible for the foodborne disease listeriosis. Despite the fact that the disease is relatively rare, it is characterized by severe symptoms and a high mortality rate (it accounts for 20–30% of the deaths of infected individuals worldwide). In most cases, ingestion of highly contaminated food (approx. 10^9^ cells) is associated with mild-to-severe febrile gastroenteritis. By contrast, in the case of high-risk individuals such as children, the elderly and immunocompromised people, the disease is characterized by sepsis, meningitidis and meningoencephalitides [1,2,3]. Pregnant women are also a highly endangered group because *L. monocytogenes* crosses the fetoplacental barrier and causes infection in the fetus, resulting in fetal death, premature birth or complications to pregnancy [4]. The infectious dose for these groups is as low as 100 to 1000 cells [1,2,3]. In general, listeriosis is the fifth most reported zoonosis in the EU. Despite strict hygiene precaution and regular food control, around 2000 people become infected in the EU annually. Around 50% of cases result in hospitalization, and 10% percent of those infected die, which is a much higher rate compared to other foodborne and zoonotic diseases [5]. *L. monocytogenes* can be subclassified into 13 known serotypes, among which 1/2a, 1/2b, 1/2c and 4b are the most common serotypes in food and are associated with 95% of all listeriosis outbreaks. However, serotype 4b alone accounts for 50% of cases [1,6,7].

Listeriosis can be treated with various antibiotics such as benzylpenicillin, ampicillin, erythromycin, meropenem, moxifloxacin, linezolid and trimethoprim-sulfamethoxazole (EUCAST, 2023). Nevertheless, antibiotic treatment cannot be applied to all patients, especially to pregnant women. In general, the best form of protection is prevention. One of the most important prophylactic approaches is to avoid the consumption of *Listeria*-contaminated food, e.g., raw meat, soft ripened cheeses and uncooked fish products [8]. However, due to the ubiquitous dispersal of *L. monocytogenes*, the risk of ingestion and disease outbreak cannot be fully excluded even in foods such as fresh vegetable or fruits [9,10]. 

Aside from the intestinal epithelial barrier, the primary defense against invasion of *L. monocytogenes* into the host’s body is the commensal intestinal microbiota. These beneficial microbes limit pathogen growth and translocation by competing for nutrients and adhesion sites on the intestinal mucosa, as well as secreting selective antimicrobial molecules. Partly due to the ongoing spread of antibiotic-resistant pathogenic strains, there remains an opportunity to support the viability and metabolic activity of the commensal intestinal microbiota with novel dietary approaches. This effort can be supported by the administration of prebiotics, which can selectively nourish beneficial microbes [11,12,13]. 

As outlined above, prebiotics are defined as substrates that are selectively utilized by host microorganisms to confer a host health benefits. The most commonly used prebiotics in the food industry or the pharmaceutical sector are mainly non-digestive nonstarch polysaccharides and oligosaccharides [14]. The most popular prebiotics are inulin, beta-glucans, fructooligosaccharides (FOSs), galactooligosaccharides (GOSs), xylooligosaccharides (XOSs), isomaltooligosaccharides (IMOs), raffinose-series oligosaccharides (RSOs) and lactulose. The best prebiotics for infants are human milk oligosaccharides (HMOs), which are found in breast milk [15,16]. Although prebiotics should only be metabolized by beneficial bacteria, it was found that even undesirable bacteria can grow on these substrates [17,18]. 

As ubiquitous organisms, *L. monocytogenes* has the capacity to survive and growth in a diverse range of natural environments, such as soil or vegetation, as a saprophyte or in the cytosol of mammalian cells as an intracellular pathogen and is able to be metabolized different substrates as sources of the main elements for its growth. Its nutrient requirements are strain-specific and may vary depending on the strain’s origin [19]. This organism requires amino acids (leucine, isoleucine, valine, histidine, arginine, glutamine and methionine) and organically bound sulfur for its growth, but it can also metabolize peptides or complex proteins [19,20]. In the absence of these substances in the environment, bacteria utilize hexose-phosphate or glycerol as alternative energy sources. In particular, bacteria apply this mechanism when acting as intracellular pathogens, when there is a lack of amino acids in the cytosol [19]. The growth is improved by various saccharides, especially glucose. *L. monocytogenes* can ferment various mono- and oligosaccharides, e.g., fructose, lactose, mannose, trehalose, cellobiose, L-rhamnose and maltose [21,22,23,24]. It is known that *L. monocytogenes* can also utilize some polysaccharides such as maltodextrin [22] and is endowed with a functional chitinolytic system and with active lytic polysaccharide monooxygenase, which are the mechanisms associated with the ability to cleave chitin and cellulose [25,26]. 

To the best of our knowledge, there are no studies focused on the ability of *L. monocytogenes* to metabolize prebiotics. Therefore, the aim of this work was to investigate the growth properties of the most common serotypes of *L. monocytogenes* in media containing different prebiotics as the sole source of energy and to evaluate the safety of the prebiotic oligosaccharides used in foodstuffs and dietary supplements with respect to the possible support of *L. monocytogenes* growth.

## 2. Materials and Methods

### 2.1. Isolation, Identification and Characterization of Strains

*Listeria* strains were isolated from foodstuffs purchased on the market in the Czech Republic and from a swab taken from a work surface in a food processing plant according to ISO 11290-1:2017 standard [27]. Subsequently, presumptive *L. monocytogenes* colonies were taken, aseptically transferred into brain–heart infusion broth (Oxoid, Basingstoke, UK) and cultivated at 37 °C for 24 h. Freshly grown isolates were checked for purity using a phase-contrast microscope (Nikon Eclipse E200LED MV RS, Tokyo, Japan) and identified using MALDI-TOF mass spectrometry (Bruker Daltonik GmbH, Leipzig, Germany) as described by Salmonová et al. [28] to the genus level. *Listeria* spp. were then identified through 16S rDNA sequencing. Briefly, genomic DNA was isolated from fresh overnight cultures using the PrepMan Ultra kit (Applied Biosystems, San Francisco, CA, USA) according to the manufacturer’s instructions. Amplification of the 16S rRNA gene was performed using fD1 [5′ AGAGTTTGATCCTGGCTCAG 3′] and rP2 [5′ ACGGCTACCTTGTTACGACTT 3′] primers [29] under the conditions described by Salmonová et al. [28]. The amplified PCR products were verified through electrophoresis on 1% agarose gel, purified with E.Z.N.A.^®^ Cycle Pure kit (Omega Bio-tek, Norcross, GA, USA) and sequenced using the Sanger method (GATC Biotech, Konstanz, Germany). Sequencing data were analyzed using the ClustalX package in BioEdit version 7.2.5 [30,31] and compared with sequences available in the GenBank nucleotide databases in NCBI [32] and EZ Biocloud [33]. The sequences were deposited in GenBank via the BankIt program at the NCBI website (https://www.ncbi.nlm.nih.gov/WebSub/?tool=genbank), accessed on 29 October 2023.

The *L. monocytogenes* serotype was determined with the slide agglutination method using commercial antisera according to the manufacturer’s instructions (Mast Group, Liverpool, UK). Pathogenicity was explored in vitro by hemolysis testing and in silico by internalin B testing. First, the hemolytic activity was detected. Cultures were spread on Columbia blood agar (Oxoid, Basingstoke, UK) supplemented with 5% sheep blood (*v*/*v*) and incubated for 24 h at 37 °C. Next, the presence of the gene for internalin B, a surface protein responsible for adhesion and invasion to the host cells, was detected via the PCR method using primers lmo2821-F [5′ TGTAACCCCGCTTACACAGTT 3′] and lmo2821-R [5′ TTACGGCTGGATTGTCTGTG 3′] [34]. 

Four hemolytic and internalin B-positive strains of different origin and serotype were selected for substrate preference testing. Strain LM1 (serotype 4b, Access. No. OR725603) was isolated from salami, LM11 (serotype 1/2b, Access. No. OR725605) from a swab from a work surface in the food industry, LM56 (serotype 1/2a, Access. No. OR725613) from raw beef meat and LM79 (serotype 1/2c, Access. No. OR725613) from vegetarian minced meat.

### 2.2. Tested Prebiotics and Cultivation Media Composition

The growth characteristics were determined in vitro in a basal medium containing 5 g/L of tryptone (Oxoid, Basingstoke, UK) and 9 g/L NaCl, which was prepared anaerobically using the Hungate technique [35]. For testing, the most commonly used prebiotics for human and livestock nutrition were selected in the form of food supplements: beta-(1,3)-D-glucan (purity of 100% yeast beta-glucans, of which >86.6% beta-(1,3)-D-glucan, Brainway Inc., Prague, Czech Republic), inulin (purity > 90%, Frutafit^®^, Sensus, Roosendaal, Netherlands), fructooligosaccharides (FOSs; purity of 95%, Nutri-Extract, Beroun, Czech Republic), galactooligosaccharides (GOSs; purity of 95%, Nutri-Extract, Beroun, Czech Republic) and 2′-fucosyllactose (purity > 94%, RAW’s, Metylovice, Czech Republic). Other frequently used prebiotics, such as lactulose (purity > 95%, Sigma-Aldrich, Saint Louis, MO, USA), and less common prebiotics, such as raffinose (purity > 99%, Sigma-Aldrich, Saint Louis, MO, USA) and stachyose (purity > 98%, Sigma-Aldrich, Saint Louis, MO, USA), were purchased in pure form. All supplements were used as received without further purification. Finally, the ability of *Listeria* strains to grow on a mixture of human milk oligosaccharides (HMOs) isolated from human milk according Rockova et al. [36] was studied. All the tested prebiotics are fully soluble in water except beta-1,3-D-glucan, which is only partially water-soluble. Concentrated stock solutions of water-soluble substrates (20 g/L) were filter-sterilized using syringe PES filters with a 0.22 μm pore size membrane (Rotilabo^®^, Carl Roth, Karlsruhe, Germany). Vials with 9 mL of basal medium were supplemented with a solution of each substrate to obtain a final concentration of 2 g/L. Due to the partial solubility of beta-(1,3)-D-glucan in water and its heat resistance [37,38], this prebiotic was added directly to the basal medium and sterilized by autoclaving at 121 °C for 15 min. An amount of residual or released glucose after autoclaving was verified spectrophotometrically using the Glucose Test (Supelco, Bellefonte, PA, USA) and measured with Reflectoquant^®^ RQflex 10 (Merck, Darmstadt, Germany). The resulting amount of glucose in the medium with beta-(1,3)-D-glucan after autoclaving was as low as 2 mg/L.

As a positive control, glucose at a concentration of 2 g/L was used as the sole carbohydrate source. Due to the incomplete purity and residual content of undefined monosaccharides in the tested substrates, a control with 0.1 g/L of glucose was also included (residual control). The medium without any carbohydrate source was tested as a negative control (basal medium). The control media were sterilized via autoclaving.

### 2.3. Testing of Bacterial Growth

Fresh overnight cultures grown under aerobic conditions at 37 °C in brain–heart infusion broth (BHI; Oxoid, Basingstoke, UK) were checked for purity and washed twice with sterile saline. The media were inoculated with bacteria suspension to a final density of 3 × 10^6^ CFU/mL and cultivated anaerobically at 37 °C for 24 h. All experiments were performed in triplicate. Bacterial growth was determined spectrophotometrically at 620 nm using a UV/VIS SPEKOL 1300 spectrometer (Analytik Jena GmbH, Jena, Germany) and calculated as a difference in optical density (OD) at the time of inoculation and after 24 h of cultivation. Since the medium with beta-(1,3)-D-glucan showed high density (OD = 0.4) in the beginning of the experiment, it was not possible to use the same method to evaluate the bacteria growth as for other substrates. Therefore, bacterial counts were evaluated with the agar plate count technique [39] using BHI agar after 24 h cultivation under aerobic conditions at 37 °C. Counts in each batch were determined in triplicate. The growth of *L. monocytogenes* strains for beta-(1,3)-D-glucan was analyzed by subtracting the initial log colony-forming unit (CFU) values per 1 mL from the final values.

The pH values of the cultivation media were measured using an HI98100 pH tester Checker^®^ Plus (Hanna instruments, Smithfield, UT, USA) at the time of inoculation and after 24 h of cultivation.

Growth curves were constructed for strains showing growth and/or pH changes (statistically significant) on the tested carbohydrates. Optical density (CFU/mL for beta-(1,3)-D-glucan), as described above, was measured in one-hour intervals during bacterial growth.

The specific growth rates were calculated for individual samples according to Rada et al. (2008) [18] using the following formula:

μ = (ln x − ln x0)/(t − t0)

where x and x0 are optical densities or CFU/mL measured within the exponential growth phase at time t and t0, respectively.

### 2.4. Enzymatic Assay

To identify the enzymes that may be responsible for substrate degradation, the tested *L. monocytogenes* strains were screened for enzymatic activity using an API-ZYM kit (BioMerieux, Marcy l’Etoile, France). Enzymes were determined according to the manufacturer’s instructions. Briefly, fresh overnight cultures grown under aerobic conditions at 37 °C in BHI broth (Oxoid, Basingstoke, UK) were checked for purity and used for testing. Bacterial suspensions were optimized to the required 5–6 McFarland density with API^®^ suspension media (BioMerieux, Marcy l’Etoile, France), and 65 μL of each inoculum was dispensed into the wells of API-ZYM strip microtubes (BioMerieux, Marcy l’Etoile, France) and incubated for 4 h at 37 °C. After incubation, a drop of ZYM A (Ref 70494) and ZYM B (Ref 70493) reagents was added. Color intensities were read according to the API-ZYM reading color scale, which ranges from 0 (negative reaction) to 5 (maximum positive reaction); approximately, scale 1 corresponds to 5 nmols, 2 to 10 nmols, 3 to 20 nmols, 4 to 30 nmols and 5 to 40 nmols or more of each API-ZYM substrate metabolized by the strains [40].

### 2.5. Statistical Analyses

The averages and standard deviations of the bacterial growth and growth rates were calculated. First, the normality of all the data sets was tested (Kolmogorov–Smirnov and Lilliefors tests). All the data were then analyzed via an ANOVA comparison followed by Tukey’s post hoc test in Statistica 12 software (StatSoft, Tulsa, OK, USA). The criterion for significance was *p* < 0.05. 

## 3. Results

### 3.1. Utilization of Water-Soluble Prebiotics

The results of *Listeria* strain growth on water-soluble prebiotics are summarized in Table 1. The growth of all tested strains in the positive control (2 g/L glucose) was significantly (*p* < 0.05) higher than on the tested carbohydrates. Slightly increased bacterial densities, which were significantly (*p* < 0.05) higher than in the negative control (no saccharides), were observed on inulin, on FOSs and in the residual control (0.1 g/L glucose). Slightly increased density was also observed in GOSs for the LM11 strain. For other strains, the density values in GOSs were similar to both negative and residual control. Other prebiotics were not utilized, and the optical density values of all strains were similar to the negative control. Strain-specific utilization was found for GOSs, stachyose and the mixture of HMOs. *Listeria* growth in the negative controls was also strain-specific.

### 3.2. Utilization of beta-(1,3)-D-Glucan (Partially Water-Soluble Prebiotic)

The growth of *L. monocytogenes* on beta-(1,3)-D-glucan is summarized in Table 2. The bacterial cell counts in media containing beta-(1,3)-D-glucan were in line with counts determined in the positive control. The growth in the negative control was significantly (*p* < 0.05) lower than on the above-mentioned purchased media.

### 3.3. Changes in pH Values in the Fermentation System

Bacterial growth was accompanied by a decrease in the pH of the culture medium (Table 3). The initial pH value was 6.9 in all batches. In the positive control and beta-(1,3)-D-glucan batches, the pH decreased to 5.7. The pH decreased to 6.3 in the inulin experiments and to pH 6.6 in the residual control, FOSs and GOSs. The values of pH did not change in media with saccharides, where bacterial growth was not detected.

### 3.4. Growth Curves and Rates

**Figure 1:**
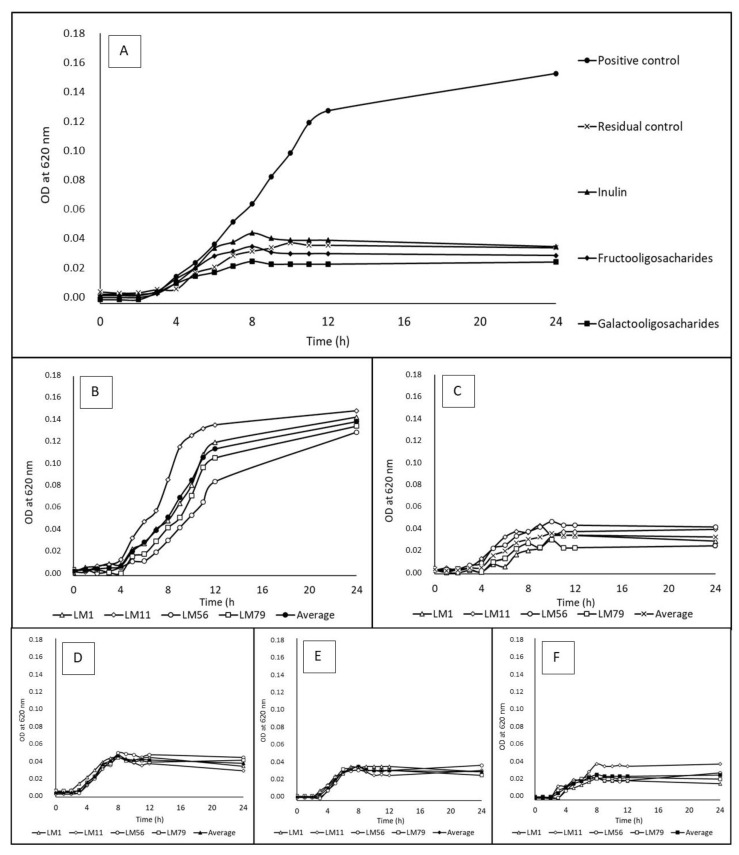
The growth curves of *Listeria monocytogenes* strains cultivated on water-soluble prebiotics (2 g/L). All data for individual strains are averages from triplicates. Positive control = 2 g/L glucose. Residual control = 0.1 g/L glucose. OD = optical density. (**A**) Data show average of 4 strains: LM1 (serotype 4b), LM11 (serotype 1/2b), LM56 (serotype 1/2a) and LM79 (serotype 1/2c). (**B**) The growth of individual strains in the positive control (2 g/L glucose). (**C**) The growth of individual strains in the residual control (0.1 g/L glucose). (**D**) The growth of individual strains on inulin (2 g/L). (**E**) The growth of individual strains on fructooligosaccharides (2 g/L). (**F**) The growth of individual strains on galactooligosaccharides (2 g/L).

Growth curves were constructed, and specific growth rates were calculated for the positive control, residual control, beta-(1,3)-D-glucan, inulin, FOSs and GOSs. The growth curves during the cultivation of *L. monocytogenes* on water-soluble prebiotics are shown in Figure 1. The specific growth rates are summarized in Table 4. Although the same specific growth rates of *L. monocytogenes* on the prebiotics and controls were found, the maximal optical density value was significantly higher in the positive control than in the prebiotic case. The growth curves show that for bacteria cultured on a medium with the residual control, inulin, FOSs and GOSs, the exponential phase lasted around 3 h, and growth was terminated at OD values between 0.028 and 0.047. A longer exponential phase (8 h) was observed during the cultivation in the positive control, where the growth terminated at OD 0.128. As in the case of bacterial growth ability, strain-specific growth rates were found for the residual control, inulin, FOSs and GOSs in this case.

**Figure 2:**
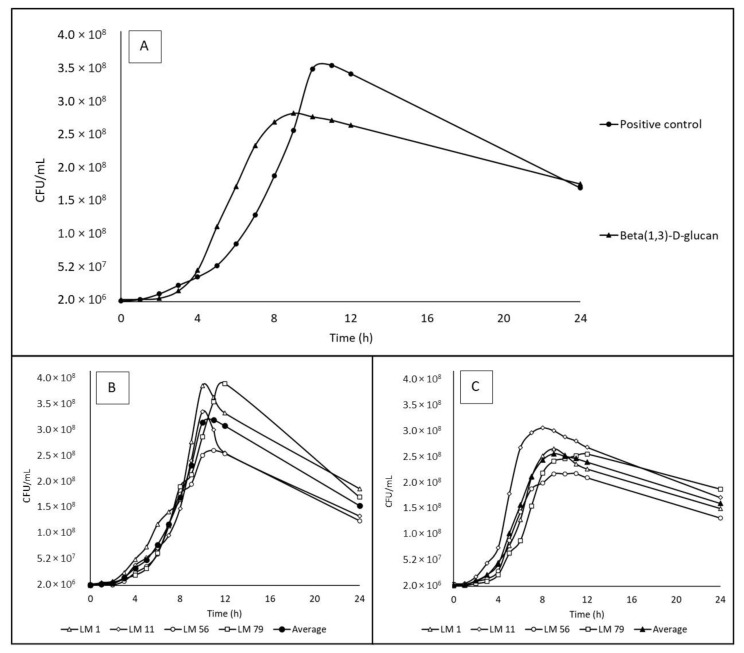
The growth curves of *Listeria monocytogenes* strains on beta-(1,3)-D-glucan (partially water-soluble prebiotic) and the positive control. All data for individual strains are averages from triplicates. CFU—colony-forming units. (**A**) Data show the mean of 4 strains: LM1 (serotype 4b), LM11 (serotype 1/2b), LM 56 (serotype 1/2a) and LM 79 (serotype 1/2c). (**B**) The growth of individual strains in the positive control (2 g/L glucose). (**C**) The growth of individual strains on beta-(1,3)-D-glucan (2 g/L).

The growth curves of bacteria cultivated in media with the positive control and beta-(1,3)-D-glucan are presented in Figure 2, and the calculated specific growth rates are shown in Table 5. No statistically significant difference (*p* ≥ 0.05) was found for the specific growth rates obtained for these two media, although the shapes of the growth curves were different with a maximal count of 3.5 × 10^8^ CFU/mL after 9 h of cultivation in the positive control and 2.8 × 10^8^ CFU/mL after 8 h of cultivation in media with beta-(1,3)-D-glucan.

### 3.5. Enzymatic Activities

The enzymatic activities of all examined *L. monocytogenes* isolates are shown in Table 6. All isolates were positive for esterase, acid phosphatase, naphtol-AS-BI-phosphohydrolase and β-glucosidase. Negative responses were observed consistently for lipase, cystine aminopeptidase, alkaline, phosphatase, chymotrypsin, N-acetyl glucosaminidase, valine aminopeptidase, trypsin, alpha-galactosidase, beta-galactosidase, beta-glucuronidase, alpha-mannosidase and alpha-fucosidase. Variable responses were noted for esterase-lipase, leucine aminopeptidase and alpha-glucosidase.

## 4. Discussion

Prebiotic saccharides should selectively stimulate the growth of health-promoting (probiotic) bacteria, suppress the occurrence of pathogenic bacteria and confer benefit(s) upon host health [14,17]. However, it has been noted that some prebiotics can be utilized by undesirable bacteria as well [17,18]. *Listeria monocytogenes*, as a foodborne pathogen, can also occur in the digestive tract, where it can use various nutrients for its growth, including several specific saccharides [19,20]. The ability of bacteria to utilize different saccharides depends on the bacteria’s genetic predisposition [19,41,42]. In general, it is known that *L. monocytogenes* has a large number of transporter genes devoted to saccharide transport through the phosphoenolpyruvate-dependent phosphotransferase systems, particularly beta-glucosides [43]. To the best of our knowledge, there are no studies focused on the ability of *L. monocytogenes* to metabolize prebiotics. Therefore, the aim of this study was to evaluate the growth of the most common *L. monocytogenes* serotypes on several prebiotic supplements with a purity of 90–100%, depending on the product, in order in order to better understand potential unintended consequences of prebiotic supplementation on gut pathogen–host interactions.

The results showed that lactulose, raffinose, stachyose, 2′-fucosyllactose and the mixture of HMOs were unable to promote *L. monocytogenes* growth, and even residual substrates present in the tested supplements did not affect the results. A slight increase in cell numbers and changes in pH values were observed for supplements containing inulin and FOSs. Inulin and FOSs are prebiotics composed of linear chains of fructose units, linked by β-(2,1) glycosidic bonds, and terminated by a non-reducing sucrose end. The difference between these two prebiotics is in the length of the chain: FOSs comprise 3–7 fructose units, whereas inulin is mainly built from 10 to 60 units [11,44]. *L. monocytogenes* is capable of utilizing fructose [24], but it seems that it is not able to cleave the β-(1,2) glycosidic bond that connects individual fructose units. Supplements containing GOSs slightly promote the growth of the strain LM11. The other *Listeria* strains were similar to the negative and simultaneously to residual control. The final pH values were equivalent to the residual control for all strains. GOSs are composed of two to eight galactose units linked together with β-(1,6) glycosidic bonds and one molecule of terminal glucose connected by a β-(1,3) or β-(1,4) glycosidic bond [45]. This prebiotic saccharide does not promote the growth of *L. monocytogenes*, because galactose is not among the substrates that provide a source of carbon for *Listeria* growth [23]. The bacterial growth and specific growth rate on these prebiotic supplements were comparable to the growth in the residual control. Therefore, we assume that the growth of *L. monocytogenes* strains was caused by the presence of residual monosaccharides in the prebiotic supplements, which constituted 5% of FOS and GOS supplements and ≤10% of inulin supplements. The higher growth on inulin than on FOSs and GOSs could be affected by the higher content of monosaccharides. Strain-specific growth of *L. monocytogenes* on certain substrates is known [19,42], which was also shown in our study. Although a statistically significant strain-specific growth was observed, no comparable growth of *L. monocytogenes* strains to that in the positive control was found.

Beta-(1,3)-D-glucan was the only prebiotic supplement on which a comparable growth of *L. monocytogenes* strains to that on the positive control was found. This polysaccharide is generally considered effective and is a widely applied prebiotic, used to protect the body against various pathogens, cancer and high LDL cholesterol levels and for its antioxidant effects, which support the host’s overall health [46]. Beta-(1,3)-D-glucan belongs to the group of beta-glucans composed only from monomers of glucose that are linked by β-(1,3) glycosidic bonds [47,48]. They are the main structural components of plants and fungi and include, among others, the major biopolymer cellulose [42,43]. The beta-glucans used in this study were of *S. cerevisiae* origin and, in addition to beta-(1,3)-glucans, also contain less than 15% beta-(1,6)-glucans [46]. The results of the enzymatic tests showed that all strains were positive for the beta-glucosidase enzyme, which was also confirmed in a study by Corral and Buchanan [49]. Beta-glucosidases are a heterogeneous group of hydrolytic enzymes that cleave beta-glycosidic bonds in a wide spectrum of substrates [50]. Currently, two widely acknowledged classifications of beta-glucosidases can be used: one based on substrate specificity and the other on the structural features of beta-glucosidase. Beta-glucosidases with broad substrate specificity hydrolyze a wide range of substrates with different bonds, e.g., β(1→3), β(1→4) or β(1→6). Beta-glucosidases include, among others, beta-glucanases (glucan-beta-glucosidases), resulting in successive removal of glucose units from beta-glucans chains [51]. Unfortunately, the API-ZYM kit cannot identify specific beta-glucosidase enzymes, but according to the fermentation assay, it can be assumed that *L. monocytogenes* produce beta-glucanases and is able to cleave both the above-mentioned beta-glucans in the tested prebiotic food supplement. Also, it has previously been demonstrated that *L. monocytogenes* is able to utilize cellulose [25,26], which is structurally similar to beta-(1,3)-D-glucan, but the glucose units in the cellulose chain are linked by β-(1,4) glycosidic bonds [52]. This ability is due to the multi-enzymatic complex, called the cellulosome, that is coded by gen *CelD* [43]. The complex comprises saccharide-active enzymes, which cleave cellulose and other similar polysaccharides and, among others, include beta-1,3-glucanase, which cleaves the above-mentioned beta-1,3-D-glucans [53,54]. Considering the enzymes detected in this study and based on the available literature, it can be assumed that the beta-glucan chains are completely degraded to free glucose units followed with classic glucose metabolism, leading to the formation of lactate, acetate, formate, ethanol and carbon dioxide in anaerobic conditions [55]. The pH values in the fermentation assay for beta-(1,3)-D-glucan and glucose were similar (pH 5.67–5.73), which also indicate identical metabolites.

Compared to the growth of previously studied *Lactobacillus*, *Bifidobacterium* and commensal *Clostridium* strains in the presence of selected prebiotics, the growth of *L. monocytogenes* in this study was lower on both glucose and beta-glucan [18,56,57]. This is most likely affected by the medium composition. The media used for probiotic/commensal testing are made for fastidious bacteria and contain complex nutritional mixtures, except for their energy source. *L. monocytogenes* can utilize some of these nutrients for its growth even without the presence of saccharides; therefore, a nutrient-poor medium is required for this organism [18,56,57].

Regarding the growth curve, some differences were observed between the course of *L. monocytogenes* growth on beta-(1,3)-D-glucan and the positive control. Slightly lower cell counts on beta-(1,3)-D-glucan could be easily explained by the fact that part of the energy that could have been used for reproduction was spent on substrate cleavage. It could also be result of gradual or incomplete cleavage. The decrease in the number of viable cells on beta-(1,3)-D-glucan was slower compared to the positive control, probably due to the gradual release of glucose from the beta-glucan chain. 

There are various natural sources of beta-glucans, e.g., yeast, barley, bacteria, seaweed, fungi and dahlia tuber. They differ not only in origin but also in glycosidic bond position as well as in their other characteristics, such as solubility in water or alkalis. Along with β-(1,3)-, β-(1,4)- and β-(1,6)-glucans are also found in supplements [57,58,59]. The origin may be one of the factors that affect the ability of bacteria to metabolize beta-glucans as has been demonstrated, e.g., for *Bifidobacterium* sp. [57]. The ability of *L. monocytogenes* to cleave prebiotics also depends on the amount of substrate in the environment, because beta-1,4-glucanase is dynamic in response to the type and concentration of available saccharides [60]. In general, the environmental conditions and the amount and composition of available nutrition in the environment can affect *L. monocytogenes* metabolism, i.e., substrate preferences [19,21].

There are other types of prebiotics, such as mannooligosaccharides (MOSs), xylooligosaccharides (XOSs), isomaltooligosaccharides (IMOs) and pectic oligosaccharides (POSs), which were not included in the experiment but may be the subject of further investigation [61,62,63]. Considering that some *L. monocytogenes* strains produce alpha-glucosidase [48], it can be assumed that *L. monocytogenes* could cleave other prebiotics that are glucose oligomers with alpha-D-(1,6)-linkages such as IMOs. Conversely, since *Listeria* spp. do not produce alpha-mannosidase [49], it can be assumed that *L. monocytogenes* will not be able to cleave MOSs. The enzymatic assay shows that *L. monocytogenes* isolates also produce other enzymes, but these are not suitable for saccharide cleavage.

Regardless of whether the *L. monocytogenes* is able to utilize these prebiotics or not, effects against pathogens, such as supporting the individual’s immunity, the growth of beneficial microbes and a reduced adherence ability of pathogens in general, have been reported for these substances [64,65,66,67]. Cumulative evidence obtained from animal models and human intervention studies strongly suggest immunostimulatory effects of beta-glucans as well as effective inhibition of pathogen proliferation, reduction in adhesion to the digestive tract wall and suppression of *L. monocytogenes* infection [68,69,70]. In addition, beta-glucans are metabolized by commensal and probiotic intestinal bacteria which leads to competition for substrate. Short-chain fatty acids (SCFAs), which are formed through microbial metabolite cross-feeding, suppress *L. monocytogenes* growth and support the body’s disease defense. Health-promoting bacteria also produce other substances that act against *L. monocytogenes*, such as bacteriocins [71,72,73,74,75,76,77,78]. All these mechanisms in the gastrointestinal tract generally inhibit *L. monocytogenes* activity. The fact that *L. monocytogenes* is able to utilize beta-(1,3)-D-glucan is therefore not alarming. Finally, the question of how else prebiotics can affect the metabolism and behavior of this pathogen arises, because different saccharides have been found to affect the pathogenic potential [79,80,81,82]; biofilm formation potential [80]; and resistance to antimicrobial substances [21], heat or acids [80]. To the best of our knowledge, there are no studies focusing on these topics for the prebiotics used in our study.

In addition, the definition of prebiotics should be discussed. The first definition of prebiotics was introduced by Glenn Gibson and Marcel Roberfroid in 1995: “Prebiotics are a non-digestible food ingredient that beneficially affects the host by selectively stimulating the growth and/or activity of one or a limited number of bacteria in the colon and thus improves host health” [83]. In time, the definition of prebiotics has been revised several times. As was mentioned in the introduction, the current definition of prebiotics from 2016 is “Prebiotics are substrates that are selectively utilized by host microorganisms to confer a health benefit” [14]. The general goal is to define prebiotics as best as possible, and this topic is widely debated; particularly, the terms “selectively” or “specific” were proposed as criteria which are no longer relevant [61,84,85]. Our results also suggest that the selectivity of prebiotics is questionable. However, this study is limited to bacterial cell culture data obtained under defined condition, and the preferences of *L. monocytogenes* in the gut tract environment should be further studied. Despite the debatable terms in the definition, the important point is that prebiotics contribute to the host’s well-being.

## 5. Conclusions

Our study attempted to evaluate the ability of *L. monocytogenes* to grow on different prebiotics. The results showed that inulin, fructooligosaccharides, galactooligosaccharides, lactulose, raffinose, stachyose, 2′-fucosyllactose and a mixture of human milk oligosaccharides are not used by *L. monocytogenes* as a nutrient or energy source. Therefore, these prebiotics can be considered health-safe with respect to the possible support of *L. monocytogenes* growth. Beta-(1,3)-D-glucan was the only prebiotic utilized by *L. monocytogenes*. Therefore, the exact role of beta-(1,3)-D-glucan with respect to listeriosis development should be further studied. Even though the results showed that beta-(1,3)-D-glucan promotes the growth of *L. monocytogenes*, the available studies highlight its prebiotic properties, including suppressive effects on listeriosis.

## Figures and Tables

**Table 1:** *Listeria monocytogenes* growth on selected water-soluble prebiotic saccharides.

Strain Code	LM1	LM11	LM56	LM79	Average ± SD
Serotype	4b	1/2b	1/2a	1/2c	-
Positive control (2 g/L glucose)	0.156 ± 0.036 ^Aa^	0.140 ± 0.027 ^Aa^	0.127 ± 0.032 ^Aa^	0.130 ± 0.030 ^Aa^	0.138 ± 0.032 ^A^
Residual control (0.1 g/L glucose)	0.028 ± 0.002 ^Ba^	0.029 ± 0.001 ^BCa^	0.028 ± 0.001 ^BCa^	0.026 ± 0.001 ^BCa^	0.028 ± 0.002 ^B^
Negative control (no saccharides)	0.016 ± 0.015 ^Ba^	−0.006 ± 0.026 ^Cb^	0.008 ± 0.008 ^BCab^	0.005 ± 0.005 ^Cab^	0.006 ± 0.017 ^C^
Inulin	0.027 ± 0.006 ^Ba^	0.028 ± 0.004 ^BCa^	0.047 ± 0.013 ^Ba^	0.043 ± 0.005 ^Ba^	0.036 ± 0.012 ^B^
Fructooligosaccharides	0.026 ± 0.031 ^Ba^	0.029 ± 0.008 ^BCa^	0.034 ± 0.011 ^BCa^	0.016 ± 0.005 ^BCa^	0.027 ± 0.016 ^B^
Galactooligosaccharides	0.008 ± 0.018 ^Bb^	0.046 ± 0.006 ^Ba^	0.018 ± 0.002 ^BCb^	0.011 ± 0.007 ^BCb^	0.021 ± 0.018 ^BC^
Lactulose	0.014 ± 0.040 ^Ba^	0.010 ± 0.014 ^BCa^	−0.001 ± 0.005 ^BCa^	−0.005 ± 0.001 ^Ca^	−0.001 ± 0.010 ^C^
Raffinose	0.004 ± 0.006 ^Ba^	0.006 ± 0.002 ^BCa^	0.004 ± 0.006 ^BCa^	−0.003 ± 0.007 ^Ca^	0.003 ± 0.006 ^C^
Stachyose	0.032 ± 0.005 ^Ba^	0.011 ± 0.009 ^BCb^	0.015 ± 0.005 ^BCb^	0.003 ± 0.005 ^BCb^	0.015 ± 0.012 ^BC^
2′-fucosyllactose	0.019 ± 0.002 ^Ba^	0.013 ± 0.003 ^BCa^	0.015 ± 0.004 ^BCa^	0.011 ± 0.006 ^BCa^	0.014 ± 0.005 ^BC^
Mixture of HMOs	0.028 ± 0.014 ^Ba^	0.010 ± 0.007 ^BCab^	0.016 ± 0.007 ^BCab^	−0.006 ± 0.010 ^Cb^	0.012 ± 0.015 ^BC^

Data are expressed as differences in OD at the time of inoculation and after 24 h of cultivation measured at 620 nm. All data for individual strains are averages from triplicates ± SD. OD—optical density. SD—standard deviation. HMOs—human milk oligosaccharides. ^ABC^—data with different superscripts differ (*p* < 0.05) in the columns. ^ab^—data with different superscripts differ (*p* < 0.05) in the rows.

**Table 2:** *Listeria monocytogenes* growth on beta-(1,3)-D-glucan (partially water-soluble prebiotic).

Strain Code	LM1	LM11	LM56	LM79	Average ± SD
Serotype	4b	1/2b	1/2a	1/2c	-
Positive control (2 g/L glucose)	1.56 ± 0.72 ^A^	1.2 ± 0.04 ^A^	0.95 ± 0.01 ^A^	1.24 ± 0.15 ^A^	1.24 ± 0.39 ^A^
Negative control (no saccharides)	0.33 ± 0.06 ^B^	0.49 ± 0.12 ^B^	0.16 ± 0.02 ^B^	0.24 ± 0.11 ^B^	0.31 ± 0.15 ^B^
Beta-(1-3)-D-glucan	1.05 ± 0.09 ^A^	1.43 ± 0.67 ^A^	0.96 ± 0.07 ^A^	1.48 ± 0.72 ^A^	1.23 ± 0.48 ^A^

Data are expressed as differences in log CFU/mL at the time of inoculation and after 24 h of cultivation. All data for individual strains are averages from triplicates ± SD. CFU—colony-forming units. SD—standard deviation. ^AB^—data with different superscripts differ (*p* < 0.05) in the columns. No statistically significant differences (*p* ≥ 0.05) in the rows were found.

**Table 3:** pH changes in batch cultures during 24 h of in vitro fermentation of different saccharides by *L. monocytogenes*.

Strain code	LM1	LM11	LM56	LM79	Average ± SD
Serotype	4b	1/2b	1/2a	1/2c	-
Positive control (2 g/L glucose)	5.70± 0.02 ^Aab^	5.68 ± 0.01 ^Aa^	5.73 ± 0.02 ^Ac^	5.71 ± 0.01 ^Abc^	5.70 ± 0.03 ^A^
Residual control (0.1 g/L glucose)	6.63 ± 0.01 ^Cab^	6.62 ± 0.00 ^Ca^	6.63 ± 0.01 ^Dab^	6.64 ± 0.00 ^Cb^	6.63 ± 0.01 ^C^
Negative control (no saccharides)	6.91 ± 0.01 ^Db^	6.90 ± 0.00 ^Da^	6.91 ± 0.01 ^Eab^	6.90± 0.01 ^Da^	6.90 ± 0.01 ^D^
Beta-(1,3)-D-glucan	5.73± 0.01 ^Ab^	5.67 ± 0.02 ^Aa^	5.73 ± 0.01 ^Ab^	5.69 ± 0.02 ^Aab^	5.71 ± 0.03 ^A^
Inulin	6.34 ± 0.02 ^Bb^	6.31 ± 0.01 ^Ba^	6.29 ± 0.01 ^Ba^	6.30 ± 0.00 ^Ba^	6.31 ± 0.03 ^B^
Fructooligosaccharides	6.63 ± 0.02 ^Cb^	6.59 ± 0.02 ^Ca^	6.60 ± 0.02 ^Cab^	6.63 ± 0.01 ^Cab^	6.61 ± 0.02 ^C^
Galactooligosaccharides	6.65 ± 0.04 ^Ca^	6.60± 0.00 ^Ca^	6.62 ± 0.02 ^CDa^	6.62 ± 0.03 ^Ca^	6.63 ± 0.03 ^C^
Lactulose	6.90 ± 0.00 ^Da^	6.89 ± 0.02 ^Da^	6.91 ± 0.01 ^Eab^	6.92 ± 0.01 ^Eb^	6.91 ± 0.02 ^D^
Raffinose	6.90 ± 0.00 ^Da^	6.91 ± 0.01 ^Dc^	6.90 ± 0.01 ^Eb^	6.90 ± 0.00 ^Da^	6.90 ± 0.01 ^D^
Stachyose	6.89 ± 0.01 ^Da^	6.90 ± 0.01 ^Da^	6.90 ± 0.00 ^Ea^	6.90 ± 0.00 ^Da^	6.90 ± 0.01 ^D^
2′-fucosyllactose	6.90 ± 0.00 ^Da^	6.90 ± 0.00 ^Da^	6.90 ± 0.01 ^Ea^	6.91 ± 0.01 ^DEa^	6.90 ± 0.01 ^D^
Mixture of HMOs	6.89 ± 0.01 ^Da^	6.90 ± 0.01 ^Dab^	6.90 ± 0.00 ^Eb^	6.90 ± 0.01 ^Dab^	6.90 ± 0.01 ^D^

Data are expressed as final pH values after 24 h of cultivation. The initial pH values in the media were 6.90 ± 0.01. All data for individual strains are averages from triplicates ± SD. SD—standard deviation. HMOs—human milk oligosaccharides. ^ABCDE^—data with different superscripts differ (*p* < 0.05) in the columns. ^abc^—data with different superscripts differ (*p* < 0.05) in the rows.

**Table 4:** Specific growth rates of *Listeria monocytogenes* on water-soluble prebiotic saccharides.

Strain Code	LM1	LM11	LM56	LM79	Average ± SD
Serotype	4b	1/2b	1/2a	1/2c	-
Positive control (2 g/L glucose)	0.42 ± 0.21 ^Aa^	0.63 ± 0.04 ^Aa^	0.49 ± 0.02 ^BCa^	0.64 ± 0.10 ^Aa^	0.55 ± 0.14 ^A^
Residual control (0.1 g/L glucose)	0.48 ± 0.07 ^Aa^	0.32 ± 0.03 ^Ba^	0.40 ± 0.08 ^Ca^	0.55 ± 0.20 ^ABa^	0.44 ± 0.14 ^A^
Inulin	0.35 ± 0.02 ^Ab^	0.63 ± 0.12 ^Aa^	0.68 ± 0.03 ^ABa^	0.51 ± 0.04 ^ABab^	0.54 ± 0.11 ^A^
Fructooligosaccharides	0.38 ± 0.10 ^Ac^	0.67 ± 0.12 ^Aab^	0.70 ± 0.09 ^Aa^	0.47 ± 0.09 ^ABbc^	0.55 ± 0.20 ^A^
Galactooligosaccharides	0.19 ± 0.05 ^Ab^	0.66 ± 0.03 ^Aa^	0.73 ± 0.18 ^ABa^	0.28 ± 0.13 ^Bb^	0.46 ± 0.19 ^A^

Data are expressed as changes in biomass concentration per hour (h^−1^). All data for individual strains are averages from triplicates ± SD. SD—standard deviation. ^ABC^—data with different superscripts differ (*p* < 0.05) in the columns. ^abc^—data with different superscripts differ (*p* < 0.05) in the rows.

**Table 5:** Specific growth rates of *Listeria monocytogenes* on beta-(1,3)-D-glucan (partially water-soluble).

Strain Code	LM1	LM11	LM56	LM79	Average ± SD
Serotype	4b	1/2b	1/2a	1/2c	-
Positive control (2 g/L glucose)	0.53 ± 0.01	0.53 ± 0.01	0.52 ± 0.01	0.47 ± 0.16	0.51 ± 0.07
Beta-(1,3)-D-glucan	0.53 ± 0.01	0.54 ± 0.01	0.53 ± 0.01	0.52 ± 0.01	0.53 ± 0.01

Data are expressed as changes in biomass concentration per hour (h^−1^). All data for individual strains are averages from triplicates ± SD. SD—standard deviation. No statistically significant differences (*p* ≥ 0.05) were found.

**Table 6:** Enzymatic profiles of tested *Listeria monocytogenes* isolates determined using the API-ZYM kit.

Strain Code	Serotype	Enzyme *						
		Esterase (C4)	Esterase Lipase (C8)	Leucine Aminopeptidase	Acid Phosphatase	Naphtol-AS-BI- -Phosphohydrolase	Alpha- Glucosidase	Beta- Glucosidase
LM1	4b	5	1	2	3	3	1	5
LM11	1/2b	4	1	2	3	2	0	4
LM56	1/2a	5	0	0	4	4	1	5
LM79	1/2c	4	0	0	4	4	0	5

Testing was performed in one repetition. The scale of the API-ZYM test was used for enzymatic quantification: 0 = no enzyme; 1 = 5 nmol; 2 = 10 nmol; 3 = 20 nmol; 4 = 30 nmol; and 5 = 40 nmol or more of metabolized API-ZYM substrate. *All isolates were negative for lipase, cystine aminopeptidase, alkaline, phosphatase, chymotrypsin, N-acetyl glucosaminidase, valine aminopeptidase, trypsin, alpha-galactosidase, beta-galactosidase, beta-glucuronidase, alpha-mannosidase and alpha-fucosidase.

## Data Availability

The original contributions presented in this study are included in this article; further inquiries can be directed to the corresponding author.
